# Integrating the Microbiome Into Infection Ecology and Evolution in Wild Animals

**DOI:** 10.1111/mec.70281

**Published:** 2026-02-26

**Authors:** Jingdi Li, Ian Will, Luís M. Silva, Tommy J. Travers‐Cook, Paradyse E. Blackwood, Kayla C. King

**Affiliations:** ^1^ Department of Zoology University of British Columbia Vancouver Canada; ^2^ Hakai Institute Campbell River British Columbia Canada; ^3^ Department of Microbiology & Immunology University of British Columbia Vancouver Canada; ^4^ Department of Biology University of Oxford Oxford UK

**Keywords:** climate change, host microbiomes, infectious diseases, parasite, parasite‐microbiome‐host interactions, wildlife

## Abstract

Parasites are a ubiquitous force in nature threatening wildlife populations and ecosystems. Interactions between hosts and their parasites are impacted by host‐associated microbiomes, which are essential for host development, physiology and immunity. We synthesise current understanding of the ecological interactions between host microbiomes and parasites, ranging from competitive to facilitative, and explore their potential evolutionary consequences for parasite virulence and transmission in the wild. We highlight recent mechanistic insights that support integrating a microbiome perspective into wildlife parasitology, with examples across diverse animal taxa including amphibians, bats, insects and corals, particularly within the context of climate change. Adopting such a holistic approach can open new avenues whereby host microbial shifts can be used to predict and mitigate infectious diseases in wild populations. Finally, we propose a conceptual framework to guide future research on microbiome‐parasite–host interactions, aiming to better reflect natural ecological complexities and advance both fundamental understanding and conservation applications.

## 1 Introduction

Parasites are ubiquitous in nature. They can harm their hosts by reducing growth, survival and reproduction (Agnew et al. 2000). Parasites can have cascading consequences for wildlife populations and wider ecosystems (Preston and Johnson 2010). The extent to which animal populations are harmed by parasites depends on the number of hosts infected, how badly infected hosts are harmed, and to what extent they are competent for onward transmission (Agnew and Koella 1997); each of these factors can be profoundly influenced by host‐associated microbiomes.

Virtually all animals and plants harbour a diverse community of microorganisms, termed the microbiome, primarily composed of bacteria, and also encompassing fungi, archaea, and viruses residing on and within the host. In the last decade, advances in sequencing technology have enabled an explosion of research into these largely unculturable microbial communities. Host microbiomes are now recognised as essential players in host development, physiology and immunity, significantly impacting host health (Malard et al. 2021; Zheng et al. 2020; Hou et al. 2022). Members of animal microbiomes have broadly shown the capacity to protect hosts against parasite infection, through host‐mediated processes (such as immune priming, behavioural changes) or/and by direct resource or interference competitions (Stevens et al. 2021; Hoang and King 2022; Jones et al. 2025; Sorbara and Pamer 2019). Conversely, microbiome‐mediated defence may be subverted by parasites, and effectively facilitate infection (Drew et al. 2021; Stevens et al. 2021). These ecological interactions between parasites and host microbiomes can impose selective pressure on parasites, thereby influencing their virulence evolution within individual hosts (Stevens et al. 2021; Smith et al. 2025; Ford et al. 2016), as well as their transmission between hosts (Berman et al. 2023).

Characterised by their large population sizes and fast metabolism, host microbiomes are inherently dynamic and can respond rapidly to biotic and abiotic changes (Li, Bates, et al. 2023; Li, Bhat, et al. 2023; Li and King 2025). Consequently, alterations of microbiome taxonomic and functional compositions may serve as indicators of parasite infection (Näpflin and Schmid‐Hempel 2018; Jaenike et al. 2010). However, the exact role of these alterations in shaping host–parasite interactions remains a central question. In healthy hosts, the microbiome typically maintains a degree of stability or homeostasis (as governed by ‘host control’, see Foster et al. 2017). Parasite invasion could disrupt this homeostatic state, resulting in microbiome *dysbiosis*. Dysbiosis has traditionally been defined as compositional changes in the microbiome, for example, reduced microbiome diversity, the absence of beneficials and blooming of pathogens (Petersen and Round 2014). But insights into the ecological causes of dysbiosis view this phenomenon as a state of weakened host control over the microbial community. This definition links dysbiosis directly to alterations in host physiological functions that regulate resource availability and govern microbial growth (Winter and Bäumler 2023). Such a loss of control can significantly influence parasite virulence and persistence, ultimately leading to adverse host health outcomes. The link between gut microbiome composition and infection progression has been extensively studied in humans (Malard et al. 2021; Jovel et al. 2018; Gomaa 2020), providing a foundation for exploring these mechanisms in wild systems.

Microbiome composition is increasingly recognised as an important predictor of infection success in specific systems. For example, Näpflin and Schmid‐Hempel (2018) demonstrated that only specific microbiome compositions allowed the establishment of *Crithidia bombi* infection in bumblebees. A similar dynamic is seen in *Drosophila neotestacea*, where populations harbouring *Spiroplasma* symbionts are resistant to infection by the parasitic nematode *Howardula aoronymphium* (Jaenike et al. 2010). Correlations between infection status and microbiome composition have been documented in wild populations of water fleas, fish and frogs (Brealey et al. 2024; Rajarajan et al. 2022; Rebollar et al. 2016; Bates et al. 2022), with further support from laboratory mice (Reynolds et al. 2015). Future work must move beyond correlation, and specifically disentangle whether observed microbial compositional shifts are drivers of resistance/susceptibility or the result of parasite‐induced disruption.

**FIGURE 1:**
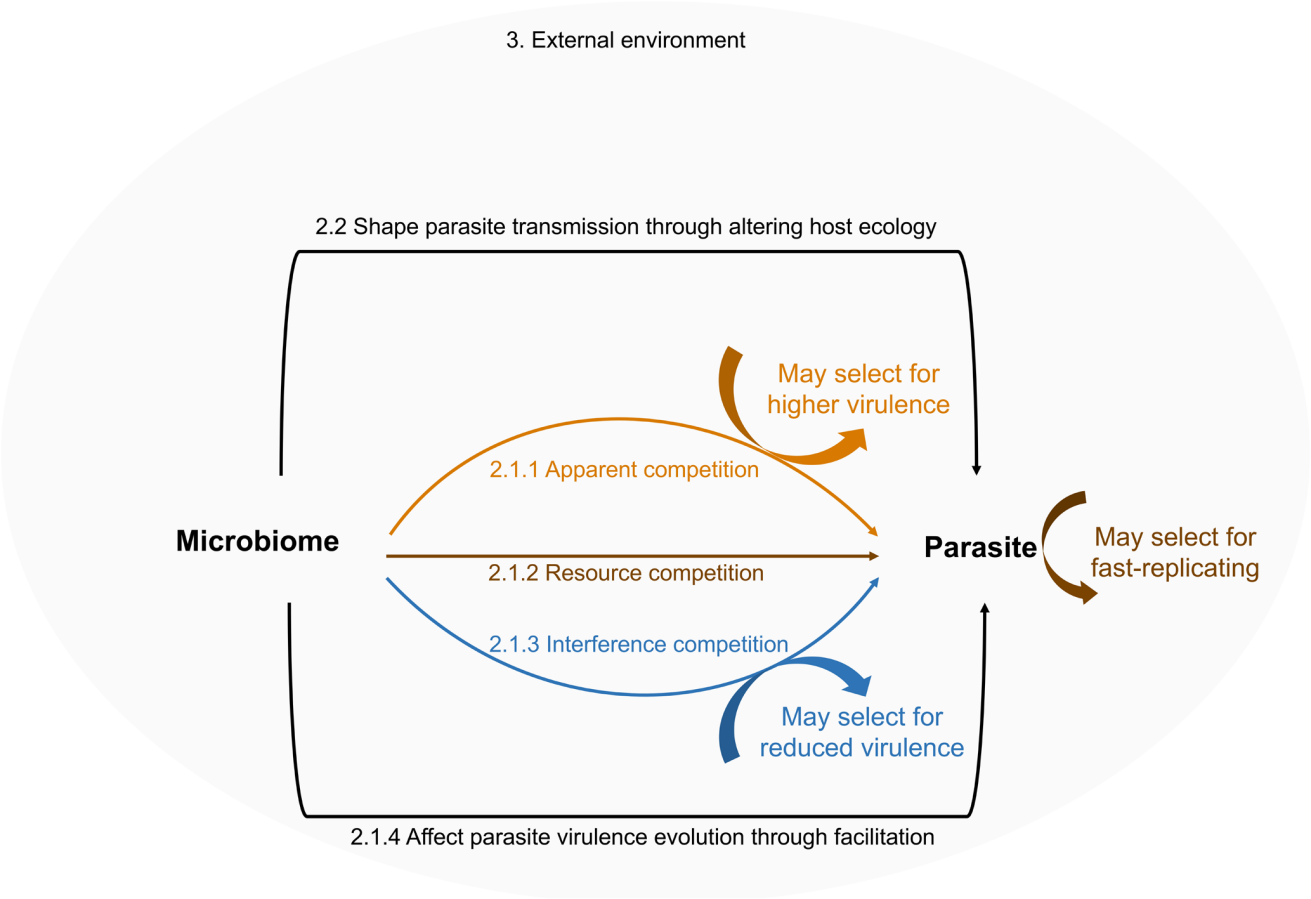
Conceptual illustration of how the host microbiome can shape parasite evolution. The microbiome can influence parasite evolution through immune‐mediated apparent competition (may drive higher parasite virulence, Section 2.1.1), resource competition (may select for fast‐replicating parasite strain, Section 2.1.2), interference competition (may select for reduced virulence, Section 2.1.3) and facilitation (Section 2.1.4). Microbiome‐driven changes in host ecology and behaviour can further shape parasite transmission dynamics (Section 2.2), while external environmental conditions modulate microbiome‐parasite interactions, and consequently parasite evolution (Section 3).

In this review, we aim to synthesise emerging evidence from wildlife (and semi‐wild) systems, demonstrating that microbiomes can play an important role in modulating infection dynamics in nature (Figure 1). We first examine the dynamics of microbiome‐parasite–host interactions and their associated evolutionary consequences within individual levels (Section 2.1). We then discuss the reciprocal relationship between host ecology and their microbiomes, analysing how within‐ and among‐individual microbial variation could shape parasite evolution (Section 2.2). To summarise potential mechanisms, we integrate insights from evolutionary hypotheses explored in lab experiments (e.g., Stevens et al. 2025; Rafaluk‐Mohr et al. 2022; Ford and King 2021; Bates et al. 2021) and theoretical modelling (e.g., Smith and Ashby 2023, 2025). Next, we explore how environmental stressors, including both single‐ and multi‐stressors linked to anthropogenic disturbances may alter host microbiomes, subsequently influencing infection (Section 3). Finally, we highlight critical gaps in our understanding of microbiome‐parasite relationships in wildlife, emphasising challenges and propose a conceptual framework for guiding future research in this field (Section 4). Understanding the impact of wild host microbiomes on infection biology is vital for predicting and mitigating parasite prevalence and transmission in nature. This knowledge may ultimately help prevent future pandemics arising from zoonotic spillover into human populations (Jenkins et al. 2015).

## 2 Evolutionary Consequences of Microbiome‐Parasite–Host Interactions

Microbiomes of animals are associated with a diverse range of host traits (Levin et al. 2021) and interface with both the host and invading parasites (Armitage et al. 2022; Vonaesch et al. 2018; Bates et al. 2022). These interactions can underpin one of two broad outcomes. First, mutualistic protection occurs when the microbiome acts in close alignment with host biology to minimise the negative impacts of disease (Hoang and King 2022; Jones et al. 2025; Sorbara and Pamer 2019). In this scenario, resident microbes benefit their hosts by modulating immunity and competing against invaders. Conversely, negative consequences arise when infection decouples host and microbiome outcomes or when parasites subvert microbiome defences (Drew et al. 2021; Stevens et al. 2021; Yu and Iatsenko 2025). Parasites may exploit microbiome‐mediated changes in the host environment or disrupt resident microbes to promote their own virulence. Just as the microbiome can bolster host immunity, parasites acting as ‘proactive invaders’ may gain a competitive advantage by triggering immune responses that they are better poised to endure than the resident microbiome (Brown et al. 2008; Brown, Fredrik Inglis, and Taddei 2009; Brown, West, et al. 2009).

Microbiome‐mediated defences inevitably drive parasite evolution and may select for traits that can further increase parasites' exploitation within and across hosts. Parasite fitness is typically influenced by a delicate balance between virulence (host exploitation and replication at the host's expense; Bull and Lauring 2014) and transmission (the ability to spread between hosts; Silva et al. 2025) (Acevedo et al. 2019; Anderson and May 1982; De Roode et al. 2008). While virulence can facilitate transmission (e.g., through coughing, diarrhoea or lesions), excessive virulence risks killing the host before transmission occurs. In contrast, benign parasites may allow for sustained infection and provide more opportunities for transmission, but may suffer from lower replication rates that limit transmission. Host ecology (Agnew and Koella 1997; Hall et al. 2007; Leggett et al. 2017) and immunity (Råberg et al. 2009; Martins et al. 2013) are well‐established drivers of parasite virulence and transmission. However, the role of microbiomes in shaping parasite evolution in wild systems remains far less understood. Current knowledge is derived primarily from theoretical models and experimental evolution of simplified systems.

### 2.1 Microbiome‐Parasite Interactions Span Antagonism‐Synergism Continuum

Within‐host interactions between resident microbiomes and invading parasites span a spectrum from antagonistic to synergistic. These interactions can confer benefits to the host by preventing or reducing disease through microbiome‐parasite competition; however, parasites can also avoid or exploit microbial defences, leading to negative outcomes. Host microbiomes can also drive the evolution of either increased (Smith et al. 2025; Hoang et al. 2024) or reduced virulence (Ford et al. 2016). The net result is highly context‐dependent. For example, re‐wilding experiments have yielded contradictory results, finding both increased (Leung, Budischak, et al. 2018; Stevens et al. 2025) and decreased parasite burdens (Knutie et al. 2017), demonstrating that simple rules do not apply. This context‐dependency highlights a critical gap in our understanding: while laboratory experiments have revealed plausible mechanisms, these mechanisms have yet to be corroborated in the wild, where hosts experience naturally assembled microbiomes, variable environments, and complex ecological interactions that are difficult to replicate in the lab. Below, we examine the mechanisms of these microbiome‐parasite interactions and their potential evolutionary consequences for the parasite.

#### 2.1.1 Apparent Competition

One of the ways resident microbiomes interact with parasites is through *apparent competition* (or immunity‐mediated competition/cross immunity). This results from microbiota influencing host development and immunity, which then mediates the infection. This influence can be long‐term, occurring well before a parasite attempts to invade, or it can happen in the moment via host immunomodulation. Across animal taxa, microbial activity appears to be critical for proper host immune development and activation. For example, reintroduction of wild mouse microbiome to pregnant laboratory mice established wild‐like microbiome communities in their offspring and resulted in enhanced survival against viral infection, likely via regulation of inflammatory signalling (Rosshart et al. 2017). Similarly, frog tadpoles reared with experimentally reduced microbiome diversity suffered greater parasite burdens later in adulthood, compared to individuals reared with microbiomes derived from natural pond water (Knutie et al. 2017). Microbial diversity in the adult frogs does not appear to predict parasite burdens, but only the long‐term impacts of the host microbiomes during early‐life development (Knutie et al. 2017). In other vertebrates such as mice and fish, microbiome communities are broadly implicated in host immune development for proper inflammatory responses, immune‐cell production and gut barrier formation (Bates et al. 2006; Benson et al. 2009; Cross 2002; Jones et al. 2025; Zheng et al. 2020). In insects, certain microbiome species and communities can enhance immunity by priming the immune system against future parasite infections (Bahia et al. 2014; Contreras‐Garduño et al. 2016; Muhammad et al. 2019; Romoli and Gendrin 2018; Song et al. 2018). Microbial interactions that consistently increase host resistance while still allowing parasite colonisation, might select for increased parasite virulence, in a dynamic analogous to the selective pressure exerted by host immune priming (Gandon et al. 2001).

#### 2.1.2 Exploitative Competition

Host microbiomes can also bolster host defences through *exploitative competition*, effectively restricting the availability of nutrient resources for the parasites (Costello et al. 2012; Freter et al. 1983). Laboratory tests using human gut microbiomes show that microbes confer colonisation resistance to parasite infection by competing for shared metabolites (Spragge et al. 2023). Increased metabolic overlap between the microbiome community and invader confers greater resistance, which is facilitated by the presence of key microbes and/or a metabolically diverse microbiome community (Spragge et al. 2023). Similarly, faecal transplants to germ‐free bumble bees, which approximate the microbiome of wild populations, reduce their burden of trypanosomatid gut parasites (Koch and Schmid‐Hempel 2011). The authors suggest that the underlying mechanisms may be competition for nutrients or space, or direct antagonisms (see below Section 2.1.3) (Koch and Schmid‐Hempel 2011). In a longitudinal field study of wild mice, a modest negative correlation between microbiome and parasite diversity suggests potential resource competition (Marsh et al. 2024). Furthermore, evidence from laboratory reared insect colonies suggests that microbiome load, rather than diversity, plays a role in defence against parasites (Hernández‐Martínez et al. 2010).

Broadly, microbes colonising animal hosts tend to have lower metabolic niche overlap than microbial communities in external environments, such as those in open water (Hester et al. 2019). If animal microbiomes accommodate relatively narrow and stable metabolic niches, it may make displacing established resident microbes difficult for parasites. Specifically, genomic analyses of human gut microbiomes suggest that more closely related microbial species tend to increase in cooperative traits like siderophore production (Simonet and McNally 2021). Iron scavenging via siderophore secondary metabolites to acquire ‘public goods’ from host resources can promote cooperation among microbiome members and potentially mediate competition with parasites (Kramer et al. 2019; Schalk 2024). Experiments competing single microbiome species with parasites in vivo have linked siderophores and iron scavenging to microbial competition in the gut (Deriu et al. 2013; Ford et al. 2016).

The competition over limited host resources can select for fast‐replicating, and therefore, more virulent parasite strains. Laboratory studies have shown that more virulent strains might be better equipped to outcompete the microbiomes for access to nutrients (Ford et al. 2016). Also, parasites evolved among certain microbiomes may be more virulent as parasite growth (i.e., host exploitation) accelerated (Rafaluk‐Mohr et al. 2022). However, this increase in virulence can carry an evolutionary cost to transmission, if the resulting host exploitation reduces host survival and shortens the transmission window (Silva et al. 2025; Silva and Koella 2024). In wild systems, these dynamics may contribute to observed variation in parasite virulence across populations exposed to different microbiome compositions or resource environments.

#### 2.1.3 Interference Competition and Direct Antagonism

Microbiomes can engage parasites via *interference competition*, a process known as antagonistic allelopathy or microbial warfare, by producing toxins and antimicrobials. This defence mechanism is documented across a wide array of hosts. For example, skin microbes isolated from wild amphibians suppress growth of chytrid (a deadly fungal parasite) in vitro (Harris et al. 2006; Park et al. 2014) and in vivo (Harris et al. 2009; Muletz‐Wolz, Almario, et al. 2017), likely due to the secretion of anti‐fungal compounds. The effectiveness of this inhibition, however, can vary based on parasite genotype and environmental factors like temperature (Muletz‐Wolz, DiRenzo, et al. 2017). Similarly, bacteria isolated from the preening (uropygial) gland from wild and captive birds reduced growth of microbial parasites when tested in laboratory cultures (Bodawatta et al. 2020). Bacterial isolates from outdoor honeybees (Evans and Armstrong 2006) or mosquitoes (Bahia et al. 2014; Cirimotich et al. 2011; Romoli and Gendrin 2018) also protect their hosts from infection. Wild tuberculosis‐free boar microbiomes harbour more abundant ‘probiotic’ species compared to those in diseased populations, and laboratory tests suggest this microbiome species mediate defensive antimicrobial production and immune stimulation (Bravo et al. 2022).

Interference competition between microbiomes and parasites may select for parasites that can either resist microbiome‐produced toxins or evolve toxin‐based strategies to directly kill and overcome their microbial counterparts, with the latter predicted by theories (Brown, Fredrik Inglis, and Taddei 2009; Brown, West, et al. 2009). Supposedly, resistance comes at a cost to the parasite; we might expect reduced virulence, assuming a trade‐off between replication (often proportional to virulence) and resistance to the toxin (Armitage et al. 2022).

#### 2.1.4 The Role of Parasite Counteraction and Microbiome Facilitation

While microbiomes play key roles in host defence, parasites have evolved sophisticated ways to counteract these protective effects (Jones et al. 2025; Leung, Graham, and Knowles 2018; Sorbara and Pamer 2019; Stevens et al. 2021). And in some cases, to directly exploit microbiomes for their own benefit.

##### 2.1.4.1 Parasite Counteraction

Invading parasites must first overcome the competition with resident microbiomes detailed above. They can sidestep competition by altering their own metabolic requirements (Caballero‐Flores et al. 2020), or disrupt the resident microbiomes to promote virulent infections (Brown, Fredrik Inglis, and Taddei 2009). Evidence from the lab indicates that microbial parasites can selectively activate a biosynthetic pathway to minimise nutritional niche competition with resident microbes (i.e., amino acid synthesis) (Caballero‐Flores et al. 2020). Theory has proposed that some parasites may be adapted to both provoke and endure severe immune responses that clear resident competitors from the microbiome (Brown et al. 2008). How exactly will parasites evolve with their microbiome competitors will largely depend on who are they evolving with (e.g., nutrition niche and toxin producing abilities of their microbiome competitors). These interactions underscore the necessity of considering variation in host‐associated microbiomes when predicting parasite evolution across hosts in the wild.

##### 2.1.4.2 Microbiome Facilitation

Protection conferred by microbiomes can lead to unexpected facilitation in parasite transmission. For example, symbiotic microbes can enhance host fitness (e.g., survival) and host turnover (e.g., reproduction) or increase stress resistance, and thereby may create a more favourable environment (Bize et al. 2008) for a parasite to grow and multiply (Han et al. 2024; Shamjana et al. 2024). All these will increase the number of susceptible hosts available to be infected thus promoting parasite transmission. For some parasites, growth in a more favourable host environment may help them better survive the inter‐host environment and potentially the next host. Costa et al. (2018), for instance, showed that infective stages of *Plasmodium* developed in lipid‐rich mosquitoes led to more virulent infections in mice (Costa et al. 2018). In the mosquito *Anopheles gambiae* , members of the genus *Pseudomonas* persist in the gut microbiome across metamorphosis and confer resistance to insecticides, increasing host survival (Silva et al. 2024). These longer‐lived mosquitoes provide parasites with more time and resources to develop, therefore increasing opportunities for transmission.

Microbiomes can directly benefit the parasite through metabolic facilitation (Ng et al. 2013). Metabolic products from microbiomes may offer opportunities for cross‐feeding and create a niche for invading parasites (San Roman and Wagner 2018). Supplementation of essential nutrients, such as the provisioning of vitamin B by mosquito microbiomes, can increase loads of dengue virus compared to germ‐free hosts (Harrison et al. 2023). Parasites can also exploit microbial signals: parasites may receive nutritional cues from the microbiome to increase virulence (Sarabian et al. 2018), or rely on microbiomes as a signal for key developmental events, such as bacterial contact promoting parasitic nematode egg hatching (Hayes et al. 2010).

Microbiomes can increase parasite load through host immune regulation (Reynolds et al. 2014). For example, mosquito microbiomes may promote host tolerance for malaria *Plasmodium* parasites (Romoli and Gendrin 2018). While host tolerance can alleviate disease severity, theory predicts that conferring tolerance may allow hyper‐pathogenic parasites to establish and persist (Smith and Ashby 2023). This effect may be especially relevant in emerging disease with novel infections promoting sub‐optimal virulence (Bull and Ebert 2008).

The link between microbial presence and parasite virulence is complex. Re‐wilding laboratory mice by housing them outdoors can lead to increased burdens of parasites, compared to germ‐free individuals (Leung, Budischak, et al. 2018). Inoculating laboratory nematodes with a microbiome representative of natural communities enhanced virulence, which remained at a similar level across generations when parasites are evolved alongside the microbiome (Stevens et al. 2025). This enhanced virulence may be due to microbiome‐mediated stimulation of parasite ‘virulence regulators’, or alteration of host body conditions, immune and stress responses (Stevens et al. 2025; Will et al. 2025). The degree to which microbiome facilitation of parasites plays a significant role in establishing infection in the wild remains unclear. In below sections, we summarise evidence showing that whether the microbiome is beneficial or detrimental to the infected host will largely depend on host's underlying ecology, specific parasite threats and the environmental contexts.

### 2.2 Microbiomes Can Shape Parasite Evolution Through Altered Host Ecology and Behaviour

Hosts and their microbiomes have co‐evolved in close association, with the microbiomes playing a central role in regulating many aspects of host traits (Kolodny and Schulenburg 2020; Henry et al. 2021; Lange et al. 2023), including behaviour and mobility (Abraham and Medzhitov 2011; Grieneisen et al. 2020; Ivanov and Honda 2012; Johnson and Foster 2018; Kabat et al. 2014). This reciprocal relationship where host traits also shape their microbiomes generates substantial within‐ and between‐individual variation in microbial composition, which could shape the heterogeneity in host resistance and susceptibility across wild populations. Since these host traits directly influence the rate and the mode of parasite transmission (Barron et al. 2015; Ezenwa et al. 2016), microbiome‐mediated phenotypic changes can have substantial downstream effects on parasite evolution.

Host movement and social behaviours determine their contact with conspecifics or environmental reservoirs (Silva et al. 2025; VanderWaal and Ezenwa 2016). Host foraging behaviour (i.e., dietary selection) may also expose individuals to different parasite landscapes (Becker et al. 2018). Host microbiomes can shape these behaviours either directly—through the production of metabolites that modulate neural and hormonal pathways—or indirectly, by influencing immune or metabolic states (Kogut et al. 2020; Levy et al. 2017; Sarkar et al. 2020; Trevelline and Kohl 2022). Host microbiomes can influence other social behaviours such as mate choice and spacing between conspecifics (Arbuthnott et al. 2016; MacManes 2011; Sharon et al. 2010), which may help limit parasite transmission. Studies across fruit flies and mice have demonstrated that hosts can detect and avoid infected individuals based on cues derived from the microbiome (Beltran‐Bech and Richard 2014; Cantini et al. 2024). In *Drosophila*, Venu et al. (2014) identified the microbiome‐dependent volatiles that signal infection status and influence mating decisions (Venu et al. 2014). These avoidance behaviours reduce contact rates between infected and susceptible hosts. Conversely, parasites can manipulate the host's social behaviour. Hosts may modify foraging behaviour to support their own function (De Roode et al. 2013; Zeferino et al. 2024) or the parasite's development (Bernardo and Singer 2017; Lafferty and Shaw 2013), or may alter social contact rates, either increasing (Herbison 2017; Reichert et al. 2023) or decreasing them (Esparza‐Mora et al. 2023; Li, Bates, et al. 2023; Li, Bhat, et al. 2023; Stockmeier et al. 2023). Parasite infection can disrupt microbiome compositions to increase transmission. For example, the skin microbiome of brown bats is disturbed by *Pseudogymnoascus destructans* infection (the causative agent of white‐nose syndrome) (Ange‐Stark et al. 2023). This microbiome dysbiosis is associated with increased sociability in otherwise solitary, hibernating bats, a behavioural change that is likely to promote parasite spread (Berman et al. 2023; Hoyt et al. 2021).

Any host movement through foraging, dispersal or migration exposes both the host and their microbiomes to new environmental conditions. These conditions include shifts in humidity, temperature, diet and local microbial communities. This environmental change drives microbiome turnover: protective taxa may be lost or replaced, or new microbial communities may enhance colonisation resistance to parasites. Over time, these shifting microbial landscapes can exert selective pressure on parasites, favouring traits such as immune evasion, generalism or transmission plasticity (e.g., some parasites are able to change from vertical to horizontal transmission) (Rafaluk‐Mohr et al. 2022; Du et al. 2023). These selective forces hold particularly true for hosts that migrate or inhabit ecologically diverse environments (Sandeu et al. 2022), where their microbiomes change frequently and unpredictably.

## 3 Environmental Change Alters Microbiome‐Parasite Interactions

Host microbiomes are inherently dynamic, responding rapidly to routine ecological shifts such as diet, temperature, host age or reproduction status (Aleman and Valenzano 2019). This existing dynamism may be aggravated by anthropogenic environmental stress, including climate change, pollution and habitat loss (urbanisation). These large‐scale stressors not only modify host microbiomes but also contribute to the spread of diseases in wild populations (Trevelline et al. 2019; Weiss and Aksoy 2011; Murdock et al. 2014). Stress‐induced microbiome alteration is often characterised by weakened host control (dysbiosis) and is associated with increased host susceptibility to parasites/disease or parasite transmission (see Section 2.1.4). The heat stress‐induced mortality of Pacific oysters ( *Crassostrea gigas* ) that occurred after *Vibrio* sp. infection was due to an increase in putative bacterial parasites in the host microbiome, which has detrimental consequences for aquaculture (de Angeli Dutra et al. 2023; Green et al. 2019; Lokmer and Mathias Wegner 2015; Scanes et al. 2021). Bestion et al. (2017) found that for the lizard (*Zootoca vivipara*), climate warming (i.e., climates that are 2°C–3°C warmer) substantially reduced microbiome diversity (by 34%), which can negatively affect host survival (Bestion et al. 2017) and potentially would increase host susceptibility to parasitic infection. Conversely, microbial rapid responses may help buffer hosts against environmental stress (e.g., heat stress) and parasite infection (Eterovick et al. 2024). In infected common Coquí frogs ( *Eleutherodactylus coqui* ), increasing body temperatures across seasons is associated with increased skin microbiome diversity, which is associated with reduced parasite transmission and/or increased host resistance (Longo and Zamudio 2017). As global change intensifies, microbiome alterations may lead to unpredictable shifts in wild population disease dynamics.

Evidence across diverse systems demonstrates the impact of a single environmental stressor on host microbial dynamics (Bernardo‐Cravo et al. 2020; Cohen et al. 2020; de Angeli Dutra et al. 2023; Greenspan et al. 2020). Temperature may be the most critical factor that impacts host microbiomes (Li, Bates, et al. 2023; Li, Bhat, et al. 2023; Cohen et al. 2020; Lokmer and Mathias Wegner 2015; Moghadam et al. 2018; Sepulveda and Moeller 2020). Warming‐induced dysbiosis in the gut microbiome of *Ololygon perpusilla* tadpoles can stunt host growth (Greenspan et al. 2020). In corals, increasing temperatures cause thermal bleaching and microbiome alterations that lead to the disintegration of the coral‐algae symbiotic relationship (Bourne et al. 2008; Littman et al. 2011; Vega Thurber et al. 2020) and are associated with increased disease outbreaks (Brandt and McManus 2009; Muller et al. 2018). North American white ibis (*Eudocimus albus*; an urban bird) experience reduced gut microbial diversity and altered microbiome composition because of changes in habitat use (urban land cover, habitat loss, potential increased temperatures) and diet (anthropogenic food resources). Birds with less diverse microbiomes can have increased parasite susceptibility (Murray et al. 2020; Blackwood et al. 2025; Brans et al. 2017). Pollution, such as microplastics, perturbed the microbiome by reducing beneficial lactic acid bacteria and increasing potential pathogenic microorganisms (Proteobacteria and Vibrionales), causing inflammation in juvenile *Dicentrarchus labrax* (European sea bass) (Montero et al. 2022; Handy et al. 2023). Overall, these findings are often observational and context‐dependent. More mechanistic investigations are needed to identify clear and general influences of temperature and other persistent environmental stressors on microbiomes, as well as on the associated infection dynamics.

Wild animals are rarely impacted by a single stressor; instead, multiple stressors typically act on them and their microbiomes simultaneously. There have been a few studies examining how multiple stressors impact the microbiomes but not infection or vice versa. For example, combined exposure to increasing temperatures and nitrate pollution in European common frog ( *Rana temporaria* ) tadpoles altered the gut bacteria composition, causing reduced body condition that may in turn impact infection susceptibility (Eterovick et al. 2024). Since not all three components (host, microbiome, parasite) will react to environmental stressors in the same way, it is important to tease apart the mechanisms (Leung, Graham, and Knowles 2018). Different stressors may impact microbiome‐parasite interactions in an antagonistic, additive or synergistic way when they are combined (Marcogliese 2008; Grabner et al. 2023). With ongoing climate change, understanding the impacts of combined stressors will be critical to anticipate and mitigate infectious diseases in wild populations.

## 4 Conclusions and Future Directions

Despite recognition that host microbiomes play important roles in infection dynamics, there is a lot to explore in wild populations. We have highlighted examples of studies that use wild organisms and natural host‐associated microbiomes in the field or laboratory. However, many of the clearest mechanistic insights come from simplified and more homogenous model experimental systems (e.g., single symbiont rather than complex microbial community, lab‐adapted host organisms, etc.). This approach highlights a critical gap concerning translation of these findings to more complex and diverse natural ecosystems. In the wild, microbiomes might modulate parasite ecology and evolution in more context‐dependent ways, hinging on factors such as microbiome diversity (Näpflin and Schmid‐Hempel 2018; Mockler et al. 2018), co‐infection rates and parasite diversity (Betts et al. 2016; Johnson and Hoverman 2012), seasonal host and parasite phenology (MacDonald and Brisson 2022) and host density (Johnson et al. 2024).

**FIGURE 2:**
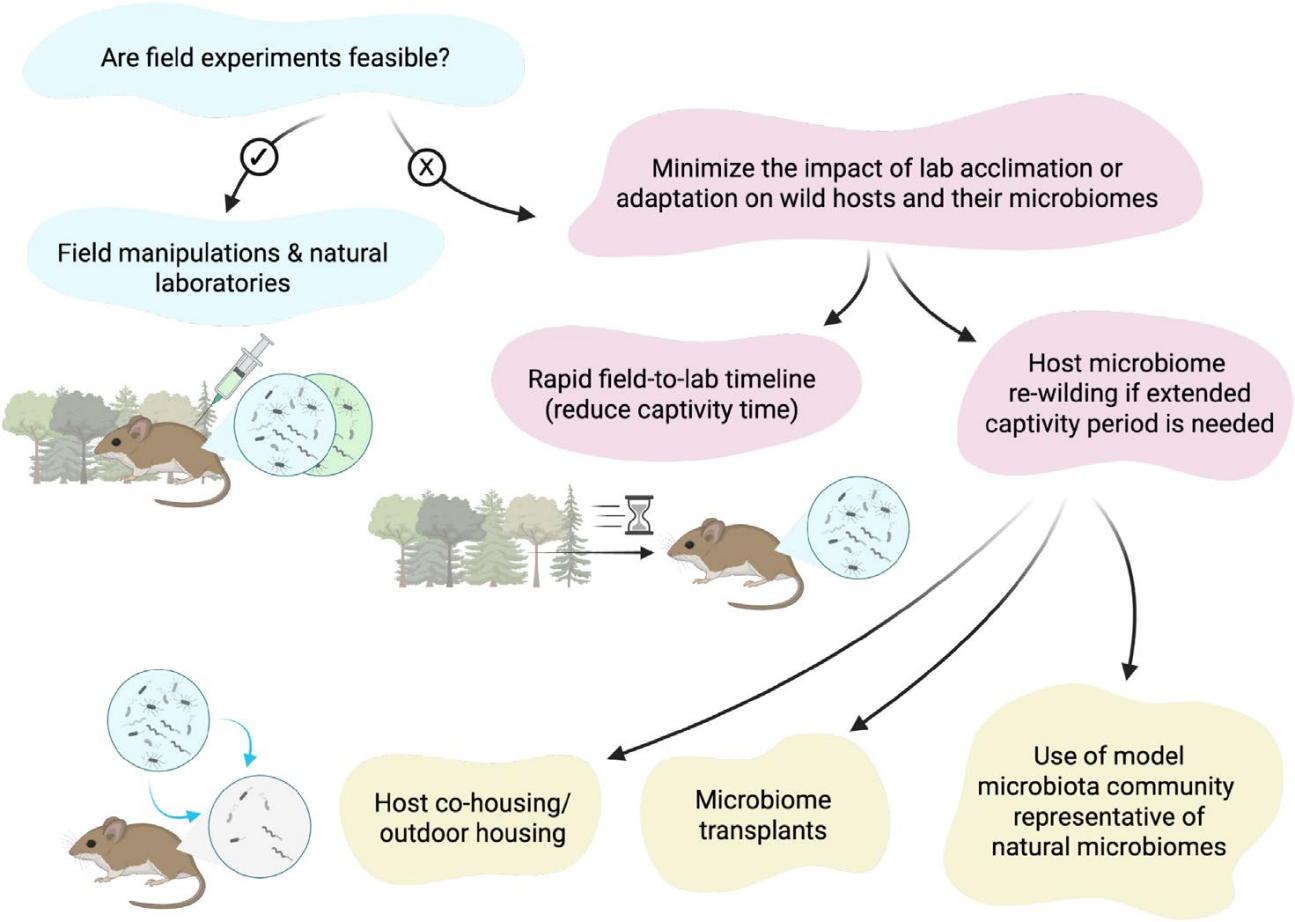
The conceptual framework for studying microbiome‐parasite–host interactions in wildlife. This framework outlines our recommended approaches, where we highlight that it is crucial to assess and minimise the impact of potential lab adaptation on wild hosts and their microbiomes. We recommend field manipulations, or a rapid field‐to‐lab timeline to reduce host captivity duration. When an extended captivity period is necessary, re‐wilding host microbiomes may be an option. This can be done through methods like co‐housing or transplants from wild hosts, outdoor housing, or the use of model microbiota communities representative of natural microbiomes. Created with BioRender.com.

Advancing the field will require prioritising development of field‐based approaches that incorporate individual‐ and population‐level longitudinal parasite tracking, microbiome profiling and ecological monitoring. By doing so, even in semi‐natural environments, it may be possible to bridge the gap between reductionist insights and ecological reality. We propose a conceptual framework to guide future research on microbiome‐parasite interactions within wildlife (Figure 2).

Field manipulation of wild populations, when feasible, offer realistic investigations closest to the natural ecology of microbiome‐parasite interactions. Multiple studies have deployed anti‐parasitic drug treatments in wild mammal and bird populations to investigate the impact on host health (Pedersen and Fenton 2015) and coinfecting parasites (Knowles et al. 2013). These experimental approaches could also be adjusted to examine how parasite reduction could affect their microbial competitors (i.e., microbiomes) within the same host. In other cases, ‘natural laboratories’ or mesocosms have helped researchers identify plausible drivers of co‐infection dynamics in nature (Halle et al. 2024). Challenges inherent to longitudinal microbiome sampling in the wild must inform our research designs. Tracking individuals often requires labour‐intensive capture‐and‐release protocols, making systems like social mammals (e.g., mice, primates) and some birds the most feasible for repeated sampling. The limitation is that wild populations tracked this way are often biassed towards healthy individuals. Consequently, tracking the transient dynamics of microbiome shifts during natural disease onset is difficult. Systems like amphibians or reptiles offer a tractable alternative. They are easier to house in controlled field mesocosms, enabling researchers to conduct infection experiments or probiotics manipulation while observing microbial and environmental variation (Bletz et al. 2013). This approach facilitates the testing of probiotics' mechanisms, on whether protection is through probiotic‐parasite competition, or indirect effects of community reconstruction and synergic interactions with resident microbes, or immune induction or a combination of them all. Understanding these mechanisms is essential to predict probiotics efficacy and parasite evolution over longer timescales.

When field manipulation is not feasible, laboratory approaches can be used, but their effects on wild host microbiomes must be carefully assessed and minimised. Some organisms lend themselves to rapid field‐to‐lab timelines or temporary captive housing, presumably minimising the shifts in their microbiomes. Outdoor apiaries of honeybees allow ready access to semi‐natural populations and quick processing of collected individuals (Evans and Armstrong 2006). Within days of collecting wild ant colonies from the field, infection experiments with fungal parasites can be performed to assess changes of host microbiomes during disease progression (Vermeulen et al. 2025). Captive rearing for two generations in bees can still maintain a diverse microbiome (Koch and Schmid‐Hempel 2011). Rearing for extended periods can lead to notable changes in microbiome composition (Kreisinger et al. 2014; Li et al. 2017; Santos Rocha et al. 2018). Alternatively, re‐wilding captive organisms can produce insights relevant to natural ecology (Bruno et al. 2024; Flies and Woods 2019; Kwon and Seong 2021; Oyesola et al. 2022; Zipple et al. 2023). Re‐wilding has been achieved with microbiome transplants or animal co‐housing (Beura et al. 2016; Oyesola et al. 2024; Rosshart et al. 2017), outdoor housing or introduction of natural substrate (Kaganer et al. 2023; Knutie et al. 2017; Leung, Budischak, et al. 2018), as well as designing model microbiota communities to mimic natural microbiomes (Dirksen et al. 2020; Oh and Rehermann 2021; Graham 2021). Notably, re‐wilding may also suffer from the fact that some microbes or parasites in the wild are uncultivable or poorly characterised, restricting our ability to manipulate or detect them in controlled experimental settings (Liu et al. 2022).

The most critical outstanding question that spans both wild observation and controlled manipulation is the need to differentiate between intrinsic microbial variations and fluctuations (driven by seasonality, diet or noise), and disease‐related changes that impact on host fitness. Answering this requires a dual approach. First, longitudinal studies must consistently collect robust host fitness metrics (e.g., disease status, reproductive output) alongside microbiome data. Then putative microbial signals identified in the field samples must be tested through experimental causality in the lab, isolating key microbes and reintroducing them to lab model hosts to confirm their direct impact on host infection resistance and fitness. This integration of wild correlation with laboratory causation is the necessary step forward.

Global change is leading to more extreme climatic events (IPCC 2023), which may destabilise host‐microbiome relationships and subvert microbiome‐mediated host defence against parasite infection. Treating the microbiome as an integral component of parasite ecology and evolution can inform more effective wildlife conservation and health strategies in a changing world.

**Nomenclature**
Parasite (or pathogen): In the context of infectious animal disease, an organism that lives in a host animal and gains fitness at a net cost to its host's fitness
Microbiota: The assembly of microorganisms from different kingdoms including bacteria, archaea, and eukaryotes
Microbiome: A characteristic microbiota occupying a given environment, including microbiota plus its distinct properties, functions, and interactions with the environment. We use microbiome here as a more inclusive term throughout the paper
Virulence: The amount of damage or harm caused by infection on their hosts
Host defence: Host responses when exposed to parasites that protect the host from being infected or reduce the detrimental effects of infection. Can be categorised into two primary types: Resistance and tolerance. Resistance is neutralisation of the parasite, and tolerance is the capacity to withstand damages caused by the parasite
Transmission: The process by which a parasite is transferred from one host to another, either directly or indirectly
Microbiome dysbiosis: Disruption of the host microbial community, often characterised by reduced microbiome diversity and weakened colonisation resistance and usually associated with negative consequences on host health
Apparent competition: Indirect microbiome‐parasite interactions mediated by the within‐host environment, where one actor loses fitness and the other's fitness can either increase or not change. Here, we take apparent competition broadly to include any host‐mediated effect, such as immunomodulation
Exploitative competition: Microbiome and parasites taking shared resources at each other's expense, such as nutrients, microbial public goods or physical space within the host
Interference competition: Direct microbiome‐parasite antagonisms such as predation or molecular warfare

## Author Contributions

**Jingdi Li:** conceptualisation (lead); writing – original draft (equal); writing – review and editing (equal); visualisation – figures (lead). **Ian Will:** conceptualisation (supporting); writing – original draft (equal); writing – review and editing (equal); visualisation – figures (supporting). **Luís M. Silva:** conceptualisation (supporting); writing – original draft (equal); writing – review and editing (equal). **Tommy J. Travers‐Cook:** conceptualisation (supporting); writing – original draft (equal); writing – review and editing (supporting). **Paradyse E. Blackwood:** conceptualisation (supporting); writing – original draft (equal); writing – review and editing (supporting). **Kayla C. King:** writing – review and editing (equal).

## Funding

This work was supported by Canada Excellence Research Chairs, Government of Canada.

## Conflicts of Interest

The authors declare no conflicts of interest.

## Data Availability

Data sharing not applicable to this article as no datasets were generated or analysed during the current study.
